# Student Socioeconomic Status and Teacher‐Student Perceptual Discrepancies of School Effort and Enjoyment

**DOI:** 10.1111/1468-4446.70035

**Published:** 2025-10-01

**Authors:** Valentina Perinetti Casoni, Katherin Barg

**Affiliations:** ^1^ School of Education University of Bristol Bristol UK

## Abstract

Congruence between teacher and student perceptions of student academic attitudes reflects positive teacher‐student relationships and enables teachers to adjust to students' needs. This study investigates discrepancies between teacher and student perceptions of student's school enjoyment and effort, and whether these discrepancies are associated with student SES. It also tests one mechanism—student visibility—that may be driving the association with student SES. We draw on representative survey data on children at the end of primary school in England and Scotland and use a residual method to compute perceptual discrepancies. We find that teachers significantly rate the effort and enjoyment of low SES students more negatively and the same attitudes for high SES students more positively compared to what the students' own reports would suggest. The association between SES and teacher‐student perceptual discrepancies remains significant even when SES‐differences in student visibility, captured through student prior ability and behaviour, are considered.

## 1 Introduction

Congruence between teacher and student perceptions of classroom and teaching aspects creates effective learning environments and can ensure that needs of all students are equally addressed (Bardach et al. 2019; Könings et al. 2014; Sonnleitner and Kovacs 2020). In contrast, discrepancies between teacher and student perceptions likely reflect low‐quality teacher‐student relationships, which in turn are negatively associated with student academic and social outcomes (Lippard et al. 2018; Longobardi et al. 2021). Teacher‐student perceptual discrepancies of student academic attitudes predict student grades (Harvey et al. 2016) and seem stratified by social group. A study by Kozlowski (2015) shows that—compared to students' own reports of their effort—teachers rate the effort of Black and Hispanic students less positively than that of White and Asian students, but this mismatch is accounted for by student socioeconomic status (SES) and ability. As teachers perceive the academic attitudes of students of lower SES more negatively and have lower academic expectations of them compared to students of higher social backgrounds (Batruch et al. 2023; Turetsky et al. 2021; Wang et al. 2018), the question arises whether there is a link between teacher‐student perceptual discrepancies and student SES.

This paper therefore investigates the relationship between student SES and discrepancies between teacher and student perceptions of the student's effort and enjoyment in school. Student academic attitudes including effort (Jin 2024; Richardson et al. 2012) and school enjoyment (Morris et al. 2021; Schukajlow and Rakoczy 2016; Valeski and Stipek 2001) are importantly related to student school success and teacher perceptions of such attitudes influence how teachers judge their students' academic abilities (Boone and Van Houtte 2013; Brookhart et al. 2016; Kelly 2008; Randall and Engelhard 2010). Student attitudes including effort and enjoyment can also be attributed to ‘non‐cognitive skills’ (Farkas 2003) which are discussed as a mediator of SES inequalities in educational achievement (Gruijters et al. 2023; Radl et al. 2024). If teachers systematically perceive the academic attitudes of low SES students more negatively than the students themselves, this could be creating learning environments and teacher‐student relationships that reinforce SES‐inequalities in school outcomes.

Our focus lies on primary school children who are 10–11 years old, an age at which teacher‐student relationships are particularly important as they influence future academic attitudes, school achievement, and relationships with teachers in secondary school (Hamre and Pianta 2001). We use data on England from the UK Millennium Cohort Study and on Scotland from the Growing Up in Scotland study. These large‐scale survey data sets provide rich, representative information on students' family background and their own reports of their attitudes towards school as well as teacher reports of the students' attitudes, thus enabling us to capture discrepancies between teacher and student perceptions. We use data on England and Scotland—two UK countries that are very similar but with differences in curriculum and relevance of standardised testing—and discuss in what ways institutional contexts may shape teachers' judgements (e.g., Geven et al. 2021). We also empirically test a mechanism that may link student SES to teacher‐student perceptual‐discrepancies, which is student visibility. Because high SES students tend to possess the cultural resources rewarded in school (Bourdieu 1966, 1977; see also Ginsberg 2023; Yılmaz et al. 2024), they may be more engaged in class and, hence, more visible in the classroom making it easier for teachers to evaluate their attitudes (see, e.g., Calarco 2011, 2014).

### 1.1 Why Congruence Between Teacher and Student Perceptions Matters

Understanding perceptual (mis‐)matches is important for several reasons. First, congruence between teacher and student perceptions of the learning environment enables teachers to adapt their behaviour to students' needs and improves effectiveness of teaching and learning (Bardach et al. 2019; Könings et al. 2014; Sonnleitner and Kovacs 2020). When teachers and students perceive the school environment similarly, this has positive effects on students' well‐being, motivation, and school connectedness (Debnam et al. 2021). Congruence in perceptions of student attitudes to schools seems also important. Harvey et al. (2016) studied the impact of discrepancies between teacher and student perceptions of student motivation—which they label teacher bias—on middle school students' final maths and English grades. In a sample of US schools with predominantly ethnic minority students from low‐income households, they find a strong association between teacher‐student perceptual discrepancies and student's final grades.

Second, an accurate teacher perception of a student may reflect a close, positive teacher‐student relationship, while inaccurate perceptions may be symptomatic of an un‐supportive or distant relationship. Warm, supportive relationships improve students' academic self‐image, motivation, engagement, achievement, and resilience while conflictual, coercive relationships are detrimental for students' academic progress and socioemotional development (Hughes and Cao 2018; Sabol and Pianta 2012). Hence, studying perceptual differences between teachers and students and how these relate to SES can tell us about a process contributing to social inequalities in teacher‐student relationships and ultimately student outcomes.

Third, congruence between teacher and student perceptions is important because when teachers grade their students, they do not only consider students' academic abilities but also their students' motivation and engagement (Baeriswyl et al. 2011; Brookhart et al. 2016; Vanlommel and Schildkamp 2018). Consequentially, inaccurate teacher perceptions of students' attitudes can translate into ‘biased’ grades, teacher decisions, and treatment of students in everyday classroom situations (Gentrup et al. 2020; Wang et al. 2018).

Finally, studying teacher‐student perceptual discrepancies helps determine whether scholars and policy makers should be cautious when relying solely either on student or teacher reports to conduct their research and make decisions (Desimone et al. 2010; Heine et al. 2002).

### 1.2 Cultural Correspondence

Student SES may be linked to discrepancies between teacher and student perceptions of students' school effort and enjoyment because teachers and higher SES students have the same understanding of what constitutes school effort and enjoyment. According to theories of social reproduction through cultural reproduction, SES differences in educational achievement are driven by ‘cultural’ differences between social classes and by the correspondence between school's culture and the culture of the middle‐classes (Bourdieu 1966; Bourdieu and Passeron 1979; Swidler 1986). It is assumed that children's socialisation within the ‘dominant’ or middle‐class makes them familiar with the required resources and behaviours, while children from more disadvantaged social classes must acquire these in school which puts them at disadvantage (Bourdieu 1966; Bourdieu and Passeron 1979; see also Ginsberg 2023; Yılmaz et al. 2024).^1^

These unequally distributed skills and habits—also termed *cultural capital* (Bourdieu and Passeron 1979) and partly attributable to the wide concept *non‐cognitive skills* (Farkas 2003)—include an understanding of what level and kind of behaviour represents effort or trying your best in school (Kozlowski 2015). Similar to what Kozlowski (2015, 44) argued for ethnic minority students, lower SES students ‘may think they are working hard, but because their cultural toolkits do not align with their teachers' expectations, teachers may disagree about students' effort’. When it comes to students' enjoyment in school, we know that enjoyment is not strongly stratified by student social background variables (Stopforth et al. 2024) but depends on a range of factors including friendships, relationships with teachers, and imagination in lesson delivery (Gorard and See 2011). However, due to cultural correspondence, it could be argued that higher SES students and teachers consider the same enjoyment driving factors (e.g., interesting lessons) while the enjoyment of lower SES students depends more on other factors such as friendships (see also De Los Reyes 2011). Students with little interest in lessons and negative attitudes to learning, for example, might be perceived as not enjoying school, while they do in fact have fun spending time with their friends in school (Willis 1977).

Another related process why student SES may be linked to differences between teachers' and students' perceptions of student's effort and enjoyment could be that teachers and students use different ‘frames of reference’ (Heine et al. 2002). Teachers might generally be drawing incorrect inferences about and misinterpreting the behaviour of students (Duckworth and Yeager 2015), but even more so if the relative social standards they use for their evaluation are systematically different from those the student employs (Heine et al. 2002). Teachers likely compare an individual student to the whole class of other students of the same age while a student will draw comparisons with their close peers as they are the ones they regularly interact with. Since students tend to have peers with similar SES backgrounds (Zwier and Geven 2023), among lower SES students this could mean that criteria to assess effort and enjoyment are considerably different to those teachers use. At the same time, among higher SES students, standards might be more aligned with the teachers' standard because their ‘cultures’ match and they use their high SES peers as reference points.

### 1.3 Student Visibility

Effort and enjoyment are student attitudes that may be difficult to assess due to limited information teachers can base their assessment on. Teachers report that they use student engagement in class to assess effort, and verbal and non‐verbal engagement are seen as ‘outward manifestation of a joyful student’ (Frenzel et al. 2018, 630). However, engagement is not always *visible* and may be an insufficient indicator of effort and enjoyment. For students who are shy and verbally engage little during class, the teacher can find it difficult to judge the students' academic attitudes and emotions (Coplan et al. 2015). Moreover, children's misbehaviour during lessons could be masking students' positive emotions and effort preventing teachers from making accurate assessments of these attitudes (Cothran et al. 2009; Sun and Shek 2012).

Since middle‐class students tend to be more likely to have the cultural resources (e.g., verbal skills) that can buffer negative effects of shyness (Coplan and Weeks 2009) and increase student confidence to engage in lessons (Calarco 2011, 2014), teachers might find it easier to correctly assess their school effort and enjoyment (Goudeau et al. 2023; Langhout 2005). Similarly, a lack of the required cultural resources might make students less confident and possibly more disruptive in lessons (Khoo and Oakes 2003). Experimental and ethnographic research in pre‐ and primary schools also shows that teachers give more opportunities for engagement to students of higher SES (Goudeau et al. 2023) and stereotype‐informed behaviour management prevents disadvantaged students from expressing themselves (Langhout 2005). Therefore, student shyness and misbehaviour—caused and strengthened by a lack of skills and abilities required and rewarded in school—may decrease the visibility of academic attitudes of lower SES students. This may reduce the accuracy of teachers' perceptions of their attitudes leading to larger perceptual discrepancies and teacher reports that may be negatively biased, if teachers have negative unconscious biases about low SES students (see next section).

### 1.4 SES‐Related ‘Biases’ in Teacher and Student Reports

While the mechanisms we have described so far explain why students' and teachers' *genuine* perceptions might not match, there are reasons to assume that teachers and students may *report* perceptions that are ‘biased’, that is, that do not correspond to their authentic views or the factual situation. If these ‘biases’—one could also interpret as ‘measurement errors’—are linked to student SES, this will influence the impact of student SES on teacher‐student perceptual discrepancies.

Teachers' unconscious biases and stereotypes about the academic attitudes and abilities of children from different SES backgrounds may influence how teachers evaluate their students' effort and enjoyment. In different countries, it has been shown that teachers tend to have more negative attitudes towards marginalised social groups than towards non‐marginalised groups (Pit‐Ten Cate and Glock 2019; Starck et al. 2020). Teachers view lower SES students as needing more support, being less likely to experience educational success in the future and having less favourable personal characteristics (Auwarter and Aruguete 2008; Browman and Miele 2024). Moreover, SES‐differences in student visibility likely reinforce the role of teacher bias because teachers' attitudes and stereotypes about a social group influence their perceptions of individual students even more when teachers have no, small, or ambiguous information about a student (Jussim 2012; Turetsky et al. 2021).

The reports of some students may also be affected by social desirability and self‐enhancement biases, which are psychological mechanisms to protect one's social reputation and self‐worth (Alicke and Sedikides 2009; Crowne and Marlowe 1964). Research on the validity of student reports of their abilities and school performance shows that especially younger and low‐performing students tend to overreport their achievement compared to administrative school records (e.g., Sticca et al. 2017; Teye and Peaslee 2015; Zimmerman et al. 2002). These findings may be due to students reporting better outcomes to be viewed more positively by others (social desirability) or believing, as an unconscious coping mechanism, that their outcomes are higher than they actually are (self‐enhancement). This motivational process might affect lower SES students' reports of their effort because achievement is related to SES (Stumm et al. 2022) and students, teachers, and parents subjectively conceive effort as associated with achievement (Stables et al. 2014). On the other hand, anti‐school or oppositional cultures might drive a process that goes in the opposite direction: although actually trying their best, low SES students might *report* low levels of effort because they are used to doing so to avoid exclusion and harassment by their peers (Coleman et al. 1961; Fordham and Ogbu 1986; Willis 1977). However, because prioritising peer group popularity is more pertinent to adolescence than primary school age (LaFontana and Cillessen 2010), we assume this factor to play a relatively minor part in students' effort reports at the end of primary school.

Students' survey responses about well‐being in different domains have also been found to be vulnerable to social desirability bias but with no links to student SES (Camerini and Schulz 2018). Therefore, the relationship between student SES and reports of enjoyment may be less affected by such biases than students' responses about their effort.

### 1.5 Hypothesis

Given the above‐described ideas about cultural correspondence, student visibility and possible biases in reports on both the student and teacher sides, we hypothesise that for higher SES students the discrepancy between teacher and student perceptions of students' school enjoyment and effort will be negligible or even ‘positive’—that is, with teachers ‘over‐rating’ students compared to the students' own reports. Conversely, for lower SES children we hypothesise that teacher perceptions will be considerably different from the students' own reports and more negative.

### 1.6 The Role of Education Systems

Recently, researchers have started to conduct country comparisons to explore the impact of cultural contexts and education systems on teacher bias (Geven et al. 2021; Olczyk et al. 2023). Following this trend, we conduct our study for England and Scotland—two UK countries with small but potentially relevant institutional differences. We believe that a brief discussion of these differences and their potential implications for the association between SES and teacher‐student perceptual discrepancies can prove useful beyond the British context because such institutional variations are widespread.

The education systems in England and Scotland have some differences that could potentially affect the role of teacher bias: the importance of exams and assessment and the curriculum. Compared to Scotland, standardised testing is more prevalent and test results have more implications in England. Test results are used to assess teacher and school performance (Sibieta and Jerrim 2021). This suggests that teachers in England have more information available about student ability, thus student ascriptive traits such as social class might have less influence on teachers' perceptions than in Scotland (see Geven et al. 2021). Moreover, prevalence of assessment often comes with more teacher accountability, which reduces biases in teacher judgements because it makes teachers focus on individual student attributes rather than ‘social categories’ (Krolak‐Schwerdt et al. 2013). In terms of curriculum, in England a traditional selection of subjects is taught, and it is well defined what contents should be covered (Sibieta and Jerrim 2021). In contrast, Scotland has a curriculum built around cross‐cutting areas of learning and teachers have considerable freedom in the selection of the content they teach. In principle the Scottish curriculum has the admirable aim to make teachers ‘agents of change’ who can freely tailor their teaching to students' needs (Priestley and Minty 2013). However, this flexibility is accompanied with little implementation guidance and led to school practice variation, confusion and pressure among teachers (Priestley and Humes 2010; Wallace and Priestley 2017). Under such conditions, criteria for assessing engagement and attitudes are likely vague and ambiguous giving more room for student SES to influence teacher perceptions. In comparison, the more regulated curriculum in England means more standardisation, which could reduce teacher assessment biases (Olczyk et al. 2023). These differences in curricula and the role of tests suggests that the impact of student SES on discrepancies between teacher and student perceptions might be somewhat smaller in England than Scotland.

## 2 Methods

### 2.1 Data and Measures

We use data from the UK Millennium Cohort Study (MCS) for England (University of London, 2022a, 2022b, 2022c, 2022d, 2022e, 2022f, 2023a, 2023) and Growing Up in Scotland study (GUS) for Scotland (ScotCen Social Research 2022a, 2022b). We use GUS data instead of the Scottish sample of the MCS because this is smaller and not containing the comparable teacher survey data. The MCS follows a representative cohort of around 19,000 children born in the UK in 2000–02 with data collected every 2 or 3 years from the age of 9 months on a range of child and parent characteristics, family life, child health, cognitive development and education. Our analysis uses wave five of the MCS, when both cohort members (CM) and their class teachers were surveyed. Thus, we focus on the end of primary school, when children aged 10/11 are in Year 6. At wave one, roughly 11,000 children were sampled in England; however, only 6224 cohort members were productive at wave five and had a valid teacher survey. We combined provided weights accounting for MCS's complex survey design and cross‐wave attrition and our own constructed weights adjusting for teacher survey‐specific attrition. After list‐wise deletion of cases with missing data on analysis variables, the achieved analytical samples are 5430 (enjoyment) and 5477 (effort).

GUS provides data on three cohorts of children. We use Birth Cohort 1 data on a representative sample of children born in 2004–05 in Scotland. This birth cohort captured children's development, family background and education on a yearly basis since the children were around 10 months old. Our analysis uses wave eight of the GUS, when both CMs and their class teachers were surveyed. Thus, we focus, roughly, on the end of primary school, when children aged 10/11 are in Primary 6. At wave one, around 5000 children were sampled in Scotland; however, only 1833 CMs were productive at wave eight and had a valid teacher survey. We used provided weights to account for both cross‐wave attrition and teacher survey‐specific attrition. After list‐wise deletion of cases with missing data on analysis variables, the achieved analytical samples are 1682 (enjoyment) and 1701 (effort).

**FIGURE 1:**
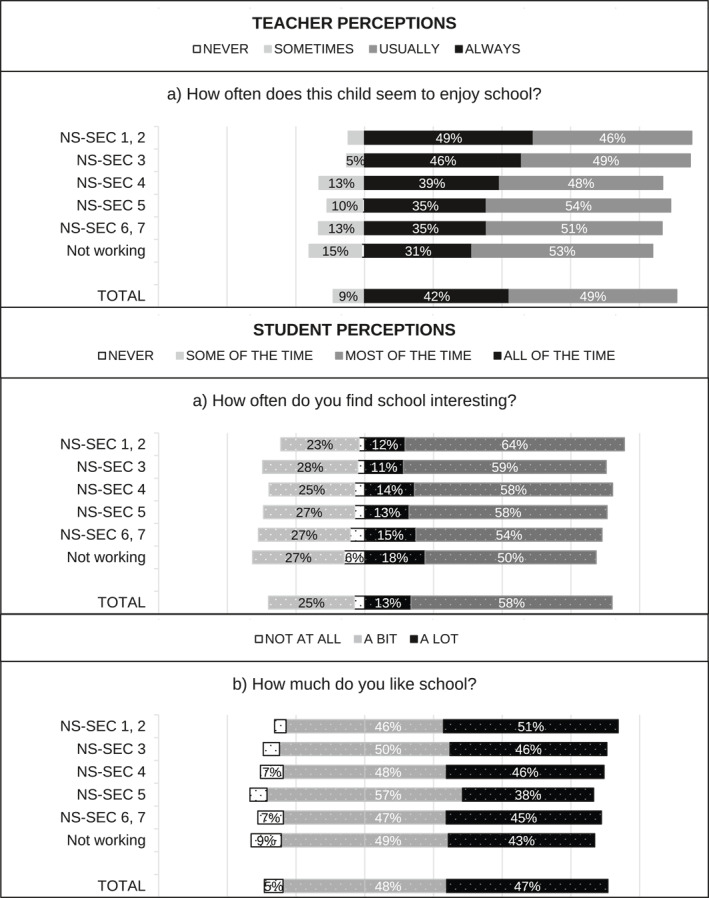
SES differences in perception of school enjoyment, England (diverging stacked bar graph).

Table 1 reports the variables used to capture teacher and student perceptions. All variables are coded on a 4‐point scale from *always/all of the time* to *never* ^2^ and standardised over the appropriate analytical sample using weights. When multiple questions were used—for example, two questions capturing student perceptions of enjoyment in England—we first created a weighted average index using the standardised items, and then we standardised the index over the appropriate sample using weights. Figure 1, 2, 3, 4 contain descriptive statistics of the perception variables before standardisation and generation of the indices. There are some differences between the measures in the English and Scottish data that need to be noted. In the English data (MCS) teacher and student perception measures tend to be more comparable than in the GUS where teachers were asked subject specific questions (e.g., enjoyment in reading, writing and numeracy) while students answered more generic questions.^3^

**TABLE 1:** Variables capturing teacher and student perceptions.

	Teacher perceptions	Student perceptions
Enjoyment		
England	How often do you think the child seem to enjoy school?	How much do you like school? How often do you find school interesting?
Scotland	How often does the child seem to enjoy: Listening & talking? Reading? Writing? Numeracy and mathematics?	I enjoy learning at school I look forward to going to school I feel happy at school How often do you find school interesting?
Effort		
England	How often does this child try their best at school?	How often do you try your best at school?
Scotland	How often does this child seem to try their best in: Reading? Writing? Numeracy and mathematics?	How often do you try your best at school?

FIGURE 2SES differences in perception of school enjoyment, Scotland (diverging stacked bar graph).

**Figure:**
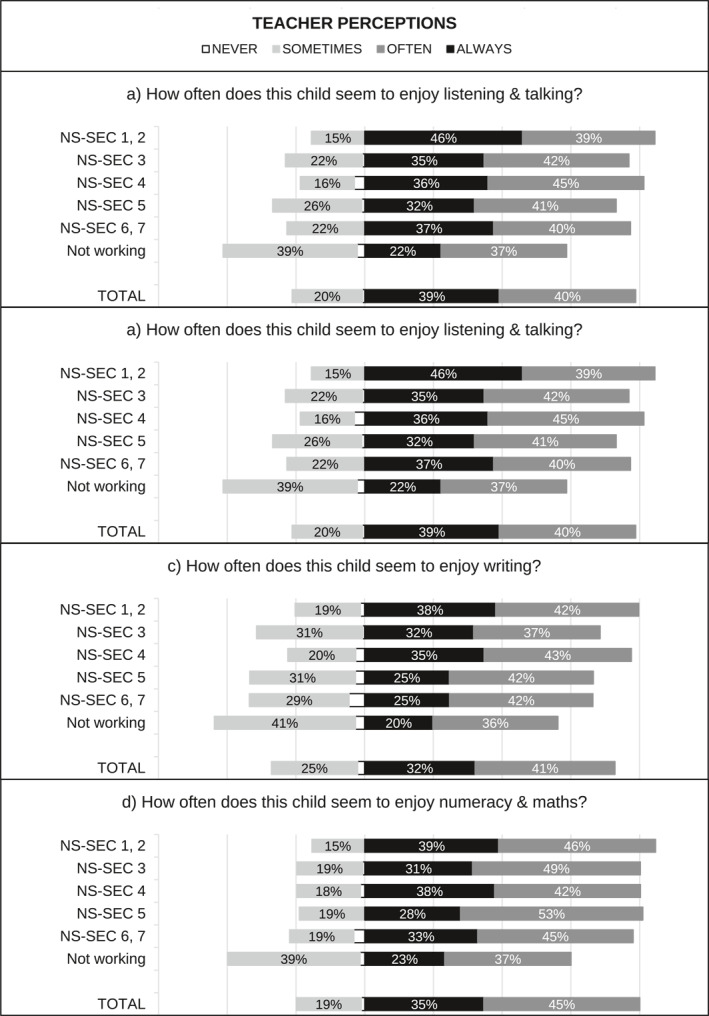

**Figure:**
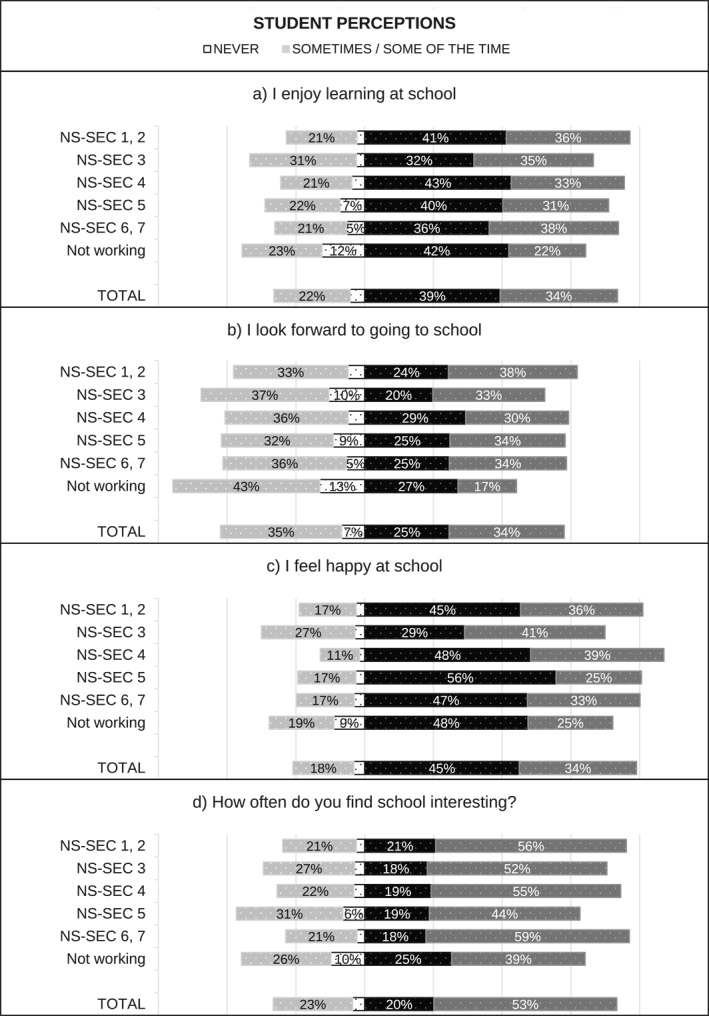

**FIGURE 3:**
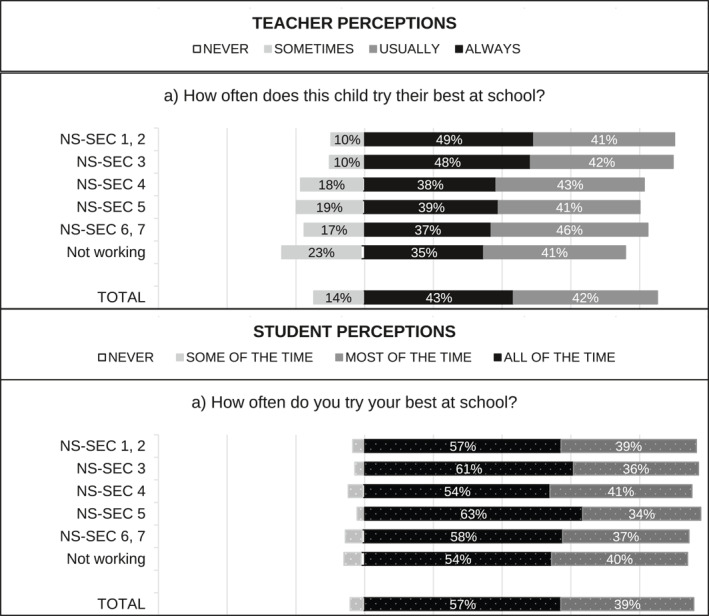
SES differences in perception of school effort, England (diverging stacked bar graph).

**FIGURE 4:**
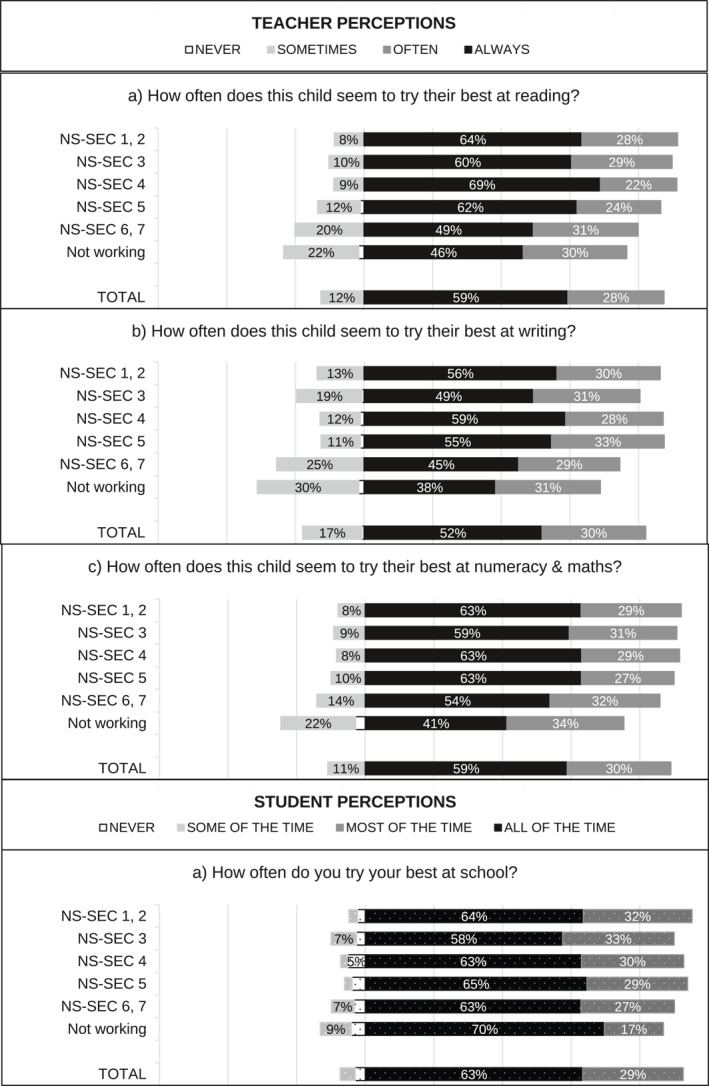
SES differences in perception of school effort, Scotland (diverging stacked bar graph).

For child SES we use the highest parental occupational status based on the UK National Statistics Socio‐economic Classification (NS‐SEC), which is the UK's official occupation‐based class scheme (Statistics 2010). It captures an individual's economic situation including income security and prospect. We use social class rather than other SES measures such as parental income or education because it is appropriate and effective to study ‘class culture’‐arguments in the context of social inequalities in education in the UK (Connelly et al. 2020; Stopforth and Gayle 2022). As described in the section ‘robustness analyses’ below, we explored alternative measures of SES such as mother's education. For SES, we differentiate six categories.

⁃ NS‐SEC 1, 2: Higher & lower managerial administrative and professional occupations,
⁃ NS‐SEC 3: Intermediate occupations,
⁃ NS‐SEC 4: Small employers and own account workers,
⁃ NS‐SEC 5: Lower supervisory and technical occupations,
⁃ NS‐SEC 6, 7: Semi‐routine & routine occupations,
⁃ Not working.^4^

To capture student behaviour in class as a factor indicating student visibility, we use the Strengths and Difficulties Questionnaire (SDQ), which is a screening questionnaire that assesses children's socioemotional and behavioural issues (Goodman 1997). SDQ has been used to predict student behaviour in primary classrooms. For example, Ribeiro et al. (2023) captured student emotional and behavioural issues using SDQ and showed that these relate to student disruptive behaviour in class. Wienen et al. (2018) employed SDQ to directly capture student behaviour in primary school. In both MCS and GUS, we use SDQ information provided by parents on emotional symptoms, conduct issues, hyperactivity & inattention, and peer relationship problems to construct the SDQ scale which we then standardised over the appropriate sample using weights. While we cannot be sure that parents' reports fully represent what is happening in the classroom, we prefer this measure over the teacher‐reported SDQ, which could introduce an endogeneity issue in our model as both the residuals and one of its predictors (teacher‐reported SDQ) may be ‘socially biased’ in the same way.

Our analysis also includes child gender and prior cognitive ability, the latter measured at the time children are aged 5 (MCS wave three and GUS wave five). Child gender may influence discrepancies between teacher and student perceptions of student attitudes because girls do better than boys across subjects (Strand 2014), enjoy school more (Stopforth et al. 2024) and tend to have closer and less conflictual relationships with their teachers (Hajovsky et al. 2017). Although not the focus of our study, such gender differences may lead to better alignment of teachers' and girls' perceptions of the girls' academic attitudes or even positive biases in teachers' perceptions, which should be considered within this study. There is a risk of collider bias when we control for prior ability because perceptual discrepancies may have a reverse impact on student achievement. We address this potential problem by making sure the temporal ordering of our independent variable (SES), mediator (achievement) and outcome (perceptual discrepancies) is appropriate (Schuler et al. 2025). More specifically we include a measure of student ability that lies temporally *before* the measure of the outcome.^5^ We capture prior cognitive ability using two British Ability Scales (BAS): BAS II Picture Similarities (capturing non‐verbal reasoning) and BAS II Naming Vocabulary (capturing expressive language skills and vocabulary knowledge) (Elliott et al. 1996, 1997). We include prior ability measures to control for cognitive, academic, and linguistic skills because, as explained in our conceptual framing above, students with these resources tend to be more engaged in lessons and, therefore, their effort and emotions will be more *visible* to teachers (see Bru 2006; Calarco 2011). We use age‐ and difficulty‐adjusted scores provided by MCS and GUS respectively and standardise these over our sample using weights (Connelly 2013). Table 2 lists descriptive statistics for all explanatory variables.

**TABLE 2:** Descriptive statistics of student SES and covariates.

	Effort		Enjoyment	
	England	Scotland	England	Scotland
	%	%	%	%
Social class				
NS‐SEC 1, 2	42.3	46.9	42.3	47.2
NS‐SEC 3	12.5	11.7	12.6	11.7
NS‐SEC 4	9.4	7.6	9.4	7.7
NS‐SEC 5	3.8	7.3	3.8	7.1
NS‐SEC 6, 7	13.1	16.6	13.1	16.5
Not working	18.9	9.9	18.8	9.8
	**Mean (sd)**	**Mean (sd)**	**Mean (sd)**	**Mean (sd)**
SDQ scale	7.9 (6.0)	7.3 (5.7)	7.9 (6.0)	7.2 (5.7)
Emotional symptoms	1.9 (2.0)	1.7 (2.0)	1.9 (2.0)	1.7 (2.0)
Conduct problems	1.4 (1.6)	1.3 (1.4)	1.4 (1.7)	1.3 (1.5)
Hyperactivity & inattention	3.2 (2.5)	3.0 (2.4)	3.1 (2.5)	3.0 (2.4)
Peer relationship problems	1.4 (1.7)	1.2 (1.7)	1.4 (1.7)	1.2 (1.7)
Prior ability				
BAS II—Picture similarities	55.0 (10.5)	58.9 (10.5)	55.1 (10.5)	58.0 (10.4)
BAS II—Naming vocabulary	54.3 (11.5)	59.2 (10.3)	54.3 (11.5)	59.2 (10.4)
*N*	5477	1701	5430	1682

*Note:* Table reports unstandardised descriptives for SDQ scale and its subscales and the two prior ability measures.

### 2.2 Analysis Technique

Analyses are conducted separately for effort and enjoyment, once for England and once for Scotland. All reported results are computed over the weighted analysis samples. After a descriptive analysis of SES stratification in teacher and student perceptions, we apply a residual approach to capture the perceptual discrepancies (Harvey et al. 2016; Gentrup et al. 2020; Hinnant et al. 2009; Madon et al. 1997): we run an OLS regression of teacher perceptions on student perceptions. The residuals of this regression capture how much an individual teacher's perception differs from the teacher perception that is predicted by the regression model. Thus, they indicate how ‘inaccurate’ each teacher's perception is and whether it is higher or lower compared to what the student's own report would suggest. Next, we run an OLS regression of these weighted standardised residuals on student SES, gender, prior ability, and SDQ score. The advantage of the residual approach over computing, for example, ‘simple’ individual differences between each student and teacher report is that the residual calculation takes into account that the distribution of teacher perceptions could be different from the distribution of student perceptions. For example, teachers' perceptions of enjoyment tend to be slightly more positive than students' own perceptions (while the opposite seems true for effort). Our approach does not presume that teachers and students use the same ‘mental scale’ or answering categories when they respond. Still, to check possible deviations in results, we ran alternative analyses using individual teacher‐student report differences (see under ‘robustness analyses’ below).

We keep our models parsimonious as we do not intend to explain the variance of the residuals (teacher‐student perceptual discrepancies) but to identify the association between student SES and the residuals, net of SES associations with student ability and socioemotional and behavioural issues, which are a proxy for student visibility.

## 3 Results

### 3.1 Descriptive Analysis

Figure 1, 2, 3, 4 show student and teacher perceptions across SES categories, before standardisation and creation of indices. The figures are diverging stacked bar graphs, which—unlike classic stacked bar graphs—place the response categories denoting our higher frequency categories (Always, Usually) to the right of a central axis and response categories representing the lower frequency options (Sometimes, Never) to the left. When the distribution of answers is read across SES groups, the overall tilt of the bars indicates whether there is an association with SES: skew to one side suggests such an association, while a roughly rectangular profile suggests none. The ‘extreme’ categories (Never and Always) are positioned nearest the centre so that any pattern in these responses is not obscured by the broader contrast between the Always/Usually‐categories on one side and the Sometimes/Never‐categories on the other side.

Overall, differences between England and Scotland seem marginal. Figures 3 and 4 show that students are more positive about their effort than teachers: rates of *never* and *sometimes/some of the time* are markedly lower in student responses than in teacher ones. Interestingly, student perceptions of effort appear less stratified by SES than teacher perceptions. Similarly, student perceptions of enjoyment are quite similar across SES groups while teacher perceptions are less so (Figures 1 and 2) and, overall, student perceptions seem less positive than teacher perceptions. Simple bivariate analysis using Spearman rank‐order correlations confirms that teacher perceptions of school effort are indeed correlated with SES (England: *ρ*(5477) = −0.1543, *p* < 0.000; Scotland: *ρ*(1701) = −0.126, *p* < 0.000) while student perceptions are not (England: *ρ*(5477) = −0.021, *p* = 0.128; Scotland *ρ*(1701) = −0.006, *p* = 0.799).^6^ Both teacher and student perceptions of school enjoyment are significantly correlated with SES, but SES associations with teacher perceptions (England: *ρ*(5430) = −0.175, *p* < 0.000; Scotland: *ρ*(1682) = −0.1965, *p* < 0.000) are larger in magnitude than with student perceptions (England: *ρ*(5430) = −0.062, *p* < 0.000; Scotland: *ρ*(1682) = −0.058, *p* = 0.017).^7^

Table 3 presents descriptive statistics for the three variables capturing student visibility—SDQ and two measures of prior ability—across student SES. In both England and Scotland, and for both attitudes' analytical samples SDQ and prior ability show clear and expected associations with student SES. Higher SES students show a higher average prior ability and lower average SDQ scores, especially in England. Although cross‐country differences in these results are small, England shows clearer ‘gradual’ SES patterns in both SDQ and prior ability resulting in larger and more consistently distributed differences, while in Scotland comparatively large negative average SDQ scores among students with non‐working parents stand out.

**TABLE 3:** Descriptive statistics of SDQ and prior ability by student SES.

Enjoyment						
	England *N* = 5430					
	NS‐SEC 1, 2	NS‐SEC 3	NS‐SEC 4	NS‐SEC 5	NS‐SEC 6, 7	Not working
SDQ scale	−0.3 (0.8)	−0.1 (0.8)	0.1 (1.0)	0.2 (1.0)	0.2 (1.0)	0.5 (1.2)
Emotional symptoms	−0.2 (0.9)	−0.1 (0.9)	0.0 (1.0)	0.1 (1.1)	0.2 (1.0)	0.3 (1.2)
Conduct problems	−0.2 (0.8)	−0.1 (0.8)	0.0 (1.0)	0.1 (1.1)	0.2 (1.1)	0.5 (1.2)
Hyperactivity & inattention	−0.2 (0.9)	−0.1 (1.0)	0.1 (1.0)	0.2 (1.0)	0.2 (1.0)	0.3 (1.1)
Peer relationship problems	−0.2 (0.9)	−0.1 (0.9)	0.0 (1.0)	0.2 (1.0)	0.1 (1.0)	0.4 (1.1)
Prior ability						
BAS II—Picture similarities	0.2 (1.0)	0.1 (0.9)	−0.0 (1.1)	−0.2 (1.0)	−0.1 (1.0)	−0.3 (1.0)
BAS II—Naming vocabulary	0.3 (0.9)	0.1 (0.9)	−0.2 (1.1)	−0.2 (1.0)	−0.2 (0.9)	−0.4 (1.0)
	**Scotland** ***N* = 1682**					
SDQ scale	−0.2 (0.9)	0.1 (1.1)	−0.1 (0.7)	−0.0 (1.0)	0.1 (1.0)	0.8 (1.3)
Emotional symptoms	−0.1 (0.9)	0.1 (1.1)	−0.1 (0.9)	0.0 (1.0)	−0.0 (0.9)	0.6 (1.3)
Conduct problems	−0.2 (0.9)	0.0 (1.1)	−0.1 (0.8)	0.1 (1.0)	0.1 (1.1)	0.5 (1.2)
Hyperactivity & inattention	−0.2 (0.9)	0.2 (1.1)	−0.1 (0.9)	−0.0 (0.9)	0.1 (1.0)	0.6 (1.3)
Peer relationship problems	−0.2 (0.9)	0.1 (1.1)	−0.1 (0.8)	−0.1 (1.0)	0.2 (1.0)	0.6 (1.3)
Prior ability						
BAS II—Picture similarities	0.1 (1.0)	0.1 (1.0)	−0.0 (1.0)	−0.4 (1.0)	0.0 (1.0)	−0.4 (0.9)
BAS II—Naming vocabulary	0.2 (0.9)	−0.1 (1.0)	−0.0 (1.0)	−0.3 (1.0)	−0.2 (1.0)	−0.4 (1.0)
**Effort**						
	**England** ***N* = 5477**					
SDQ scale	−0.3 (0.8)	−0.1 (0.8)	0.1 (1.0)	0.2 (1.1)	0.2 (1.0)	0.5 (1.2)
Emotional symptoms	−0.2 (0.9)	−0.1 (0.9)	0.0 (1.0)	0.1 (1.1)	0.2 (1.0)	0.3 (1.2)
Conduct problems	−0.2 (0.8)	−0.1 (0.9)	0.0 (1.0)	0.1 (1.1)	0.2 (1.1)	0.5 (1.2)
Hyperactivity & inattention	−0.2 (0.9)	−0.1 (1.0)	0.1 (1.0)	0.3 (1.0)	0.2 (1.0)	0.3 (1.1)
Peer relationship problems	−0.2 (0.9)	−0.1 (0.9)	0.0 (1.0)	0.2 (1.1)	0.1 (1.0)	0.4 (1.1)
Prior ability						
BAS II—Picture similarities	0.2 (1.0)	0.1 (0.9)	−0.0 (1.0)	−0.3 (1.1)	−0.1 (1.0)	−0.2 (1.0)
BAS II—Naming vocabulary	0.3 (0.9)	0.1 (0.9)	−0.2 (1.1)	−0.3 (1.0)	−0.2 (0.9)	−0.4 (1.0)
	**Scotland** ***N* = 1701**					
SDQ scale	−0.2 (0.9)	0.1 (1.1)	−0.2 (0.7)	0.0 (1.0)	0.1 (1.0)	0.8 (1.3)
Emotional symptoms	−0.1 (0.9)	0.1 (1.1)	−0.1 (0.9)	0.0 (1.0)	0.0 (0.9)	0.6 (1.2)
Conduct problems	−0.2 (0.9)	0.1 (1.1)	−0.1 (0.8)	0.1 (1)	0.2 (1.1)	0.5 (1.2)
Hyperactivity & inattention	−0.2 (0.9)	0.2 (1.1)	−0.1 (0.9)	0.0 (0.9)	0.1 (1.0)	0.6 (1.2)
Peer relationship problems	−0.2 (0.9)	0.1 (1.1)	−0.1 (0.8)	0.0 (1.1)	0.2 (1.0)	0.6 (1.3)
Prior ability						
BAS II—Picture similarities	0.1 (1.0)	0.1 (1.0)	−0.0 (1.0)	−0.3 (0.9)	−0.0 (1.0)	−0.4 (0.9)
BAS II—Naming vocabulary	0.2 (0.9)	−0.1 (1.0)	−0.0 (1.0)	−0.3 (1.0)	−0.2 (1.0)	−0.4 (1.0)

*Note:* Table reports standardised descriptives.

### 3.2 Analysis of Residuals

**FIGURE 5:**
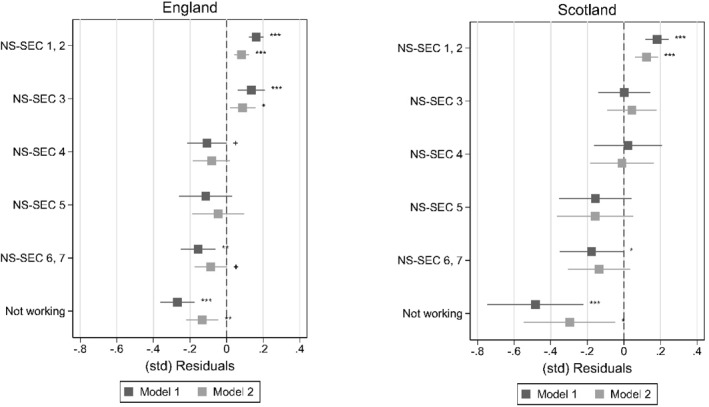
Average linear prediction of teacher‐student perceptual discrepancies by student SES, school enjoyment. +*p* < 0.10, *p < 0.05, **p < 0.01, ***p < 0.001; figures plot average linear predictions computed for M1 and M2 (see Table A3).

**FIGURE 6:**
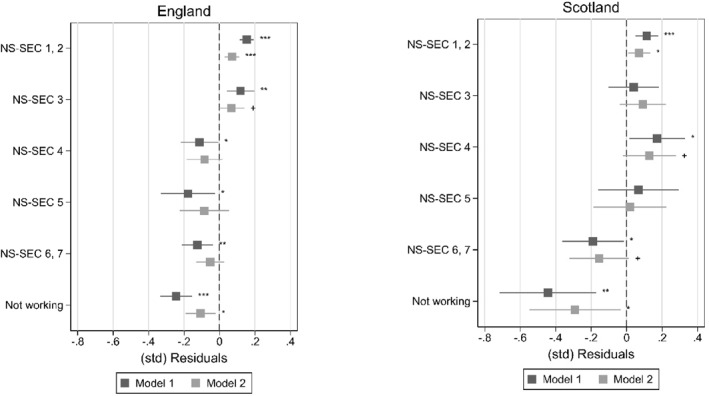
Average linear prediction of teacher‐student perceptual discrepancies by student SES, school effort. +*p* < 0.10, *p < 0.05, **p < 0.01, ***p < 0.001; figures plot average linear predictions computed for M1 and M2 (see Table A4).

As a measure of teacher‐student perceptual discrepancies, we compute residuals obtained from linear regressions of teacher perceptions on student reports (see Tables A1 and A2 in the appendix). We then run linear regressions of those (weighted and standardised) residuals on student SES and other factors. In a first model we include only student SES (M1); in a second model we add student gender, SDQ, and the two prior ability measures (M2). Figures 5 and 6 plot average linear predictions obtained from M1 and M2 for, respectively, enjoyment and effort. Tables A3 and A4 in the appendix show the regression coefficients from the models.

For both school enjoyment and school effort, M1 shows that—before including other factors—the association between SES and the residuals capturing perceptual discrepancies is significant for most SES groups, both in England and Scotland (see Figures 5 and 6). Overall, teachers perceive higher SES students more positively compared to what students' own perceptions would suggest while lower SES students are perceived more negatively. For example, the average linear prediction of school enjoyment for NS‐SEC 1, 2‐children in England is around 0.16 SD (*p* < 0.001) while it is around −0.16 SD (*p* < 0.001) for NS‐SEC 6, 7‐children and −0.27 SD (*p* < 0.001) for children with no parent in work. In Scotland, while the same overall pattern is present, for children with non‐working parents we find markedly lower predicted average standardised residuals than in England: −0.48 SD (*p* < 0.001) for school enjoyment and −0.44 SD (*p* < 0.001) for school effort. The perceptual discrepancies where teachers report more positive views for SES students than what students' own reports would predict is in line with our hypothesis. In England especially, we find teachers reporting of these more positive perceptions of school enjoyment and effort for both NS‐SEC 1, 2 and NS‐SEC 3‐students. There are hardly any variations across the two outcomes and cross‐country differences appear to be, overall, not clear or conclusive.

When student gender, socioemotional and behavioural problems (SDQ), and ability are included (M2), the magnitude and significance of the coefficients of SES‐groups decrease indicating that SES‐differences in teacher‐student differences in perceptions of student effort and enjoyment might be partly explained by SES‐differences in student visibility. It also appears that girls are viewed more positively by their teachers, both regarding school effort and enjoyment (see Tables A3 and A4 in the appendix), compared to what the students' own reports would predict.

Results presented in Tables 4 and 5 further show that the SES‐difference in the perceptual discrepancies (measured as the difference between NS‐SEC 1, 2‐students and NS‐SEC 6, 7‐students, henceforth SES‐gap) is significantly reduced in M2 in both countries and for both effort and enjoyment when ability and behaviour are controlled. For example, the SES‐gap for England when considering school enjoyment is 0.32 SD (*p* < 0.001) in M1 and 0.17 (*p* < 0.001) in M2. This decrease in the SES‐gap is statistically significant (−0.15 SD, *p* < 0.001) and roughly equal to 47% of the raw SES‐gap. When looking at school effort, results for England are similar: the raw SES‐gap is of similar size (0.28 SD, *p* < 0.001) and decreases in M2 (−0.16 SD, *p* < 0.001) by roughly 56% of the raw SES‐gap. Our results for Scotland show a raw SES‐gap from M1 that is roughly of similar size and significance for both school enjoyment and effort (0.36 SD, *p* < 0.001; 0.30 SD, *p* < 0.01). However, the gaps decrease less when variables are added in M2; the reduction is 28% for enjoyment and 26% for effort. Overall, these results suggest that student visibility may partially explain the association between SES and teacher‐student perceptual discrepancies.

**TABLE 4:** Change in SES gap across models, school enjoyment.

	England		Scotland	
	M1	M2	M1	M2
SES GAP (NS‐SEC 1, 2—NS‐SEC 6, 7)	0.32 (0.05)***	0.17 (0.05)***	0.36 (0.09)***	0.26 (0.09)**
Δ from M1		−0.15 (0.02)***		−0.10 (0.03)***
in percent		46.7		27.5

*Note:* SES gap reported as b(SE); corresponds to difference between average linear prediction of NS‐SEC 1, 2‐students and that of NS‐SEC 6, 7‐students.

+*p* < 0.10.

*p < 0.05.

^**^ *p* < 0.01.

^***^ *p* < 0.001.

**TABLE 5:** Change in SES gap across models, school effort.

	England		Scotland	
	M1	M2	M1	M2
SES GAP (NS‐SEC 1, 2—NS‐SEC 6, 7)	0.28 (0.05)***	0.12 (0.05)**	0.30 (0.09)**	0.23 (0.09)*
Δ from M1		−0.16 (0.02)***		−0.08 (0.03)*
in percent		55.7		25.7

*Note:* SES gap reported as b(SE); corresponds to difference between average linear prediction of NS‐SEC 1, 2‐students and that of NS‐SEC 6, 7‐students.

+*p* < 0.10.

^*^ *p* < 0.05.

^**^ *p* < 0.01.

^***^ *p* < 0.001.

In sum, results of our analysis show that teachers' reports are significantly more positive than what higher SES students' own reports of their effort and enjoyment would suggest. This discrepancy in perceptions remains statistically significant even when ability and socioemotional problems are held constant but is considerably reduced. This seems to show that SES‐differences in student visibility are an important driver of the SES‐impact on teacher‐student perceptual discrepancies. At the same time, our result that teachers' reports of lower SES students' attitudes are considerably more negative than what the students' own reports would predict and this discrepancy remains significant even once SES‐differences in visibility (prior ability, socioemotional problems) are accounted for could be suggesting that negative stereotypes and/or unconscious biases in relation to lower SES students and positive ones towards high SES students influence teacher perceptions when student's visibility is low, i.e., their behaviour and ability are masking their attitudes. The remaining association with SES could also be due to other mechanisms such as patterns of comparison where students judge themselves against their friends while teachers assess a student compared to the class or social desirability and self‐enhancement biases.

The comparison between England and Scotland reveals subtle differences and thus no conclusive pattern. The ‘positive bias’ for high SES students affects only students from the highest SES groups in Scotland, while in England NS‐SEC 3‐students still seem to be seen more positively by the teachers compared to their own reports. In addition, SES‐gaps in perceptual discrepancies appear slightly larger in Scotland than in England in M1, while student behaviour and visibility are more important drivers of SES differences in ‘accuracy’ of teacher perceptions in England than in Scotland. This result seems to suggest that other drivers of the impact of student SES on teacher‐student perceptual discrepancies than visibility might be more important in Scotland than in England. It also needs to be kept in mind that in the data for England teacher and student perception measures tend to be more comparable because they are both generic, while in the Scottish data the teacher variables focus on specific subjects. High SES students and girls tend to do better and believe they are better in language subjects than low SES students and boys (e.g., Sullivan 2009) and, as discussed in the theoretical section, achievement and perceptions of achievement are linked to student effort and enjoyment. Therefore, in Scotland, the reading‐ and writing‐focused measures may capture the attitudes of high SES students and girls better than the students' general ones leading to teacher‐student perceptual discrepancies that are different to those captured with the English data. This could be leading to an underestimation of country‐differences in the associations between SES (and gender) and the perceptual discrepancies.

#### 3.2.1 Robustness Analyses

We ran a set of robustness analyses of which results are reported in Supporting Information S1. Using mother's education as an alternative SES‐measure leads to a very similar pattern of results, although mother's education tends to be a bit more strongly related to the perceptual discrepancies than the NS‐SEC variable. However, as pointed out above, we settle for social class because literature suggests it to be useful for analyses of ‘cultural’ processes. We also ran analyses in which teacher‐student perceptual discrepancies are computed as ‘simple’ difference between each student and teacher report instead of using the residual method. We find very similar relationships with SES but of slightly smaller strength and, partly, with lower levels of statistical significance. Finally, we run robustness analyses with models not including gender in order to check whether including gender, prior ability and SDQ simultaneously (as shown above) affects our test of the student‐visibility mechanism. The results are very similar to those presented above indicating that the joint inclusion of the variables is not problematic.

## 4 Discussion and Conclusion

We find that teachers perceive enjoyment and effort of higher SES students more positively and those of lower SES students more negatively than the children's own perceptions would suggest. The associations between student SES and these perceptual discrepancies are partly explained by SES‐differences in student visibility, which was operationalised through parents' reports of children's socioemotional problems and prior ability measured through standardised tests. This seems to be indicating that SES‐related behaviour, skills, and academic achievement mask children's attitudes making it more difficult for teachers to accurately evaluate them and creating conditions under which teachers' unconscious biases affect their perceptions. The remaining association with SES may be driven by other mechanisms including teachers' unconscious biases, student social desirability or self‐enhancement biases, or SES‐related differences in ‘frames of reference’.

The comparison between Scotland and England reveals only little differences between the two countries. We find that student SES is associated with discrepancies in perceptions in both education systems, but that prior ability and socioemotional difficulties explain more of the SES‐influence on perceptual discrepancies in England than in Scotland. This may suggest that behaviour and ability have a comparatively stronger influence on teacher perceptions in England. The English system placing more emphasis on standardised testing could be inciting teachers to pay particular attention to student ability, especially when evaluating student effort. Moreover, at least at the time our analysis focuses on, legal frameworks in England tended to stress the power of schools to control and discipline students while the government in Scotland put more emphasis on relationships and acknowledging students' views when it comes to addressing misbehaviour (Ball et al. 2011; Macleod 2014).

Our study contributes to scholarship concerned with social inequalities in academic attitudes and feelings—student characteristics that can be assigned to the broad concept of ‘non‐cognitive skills’—and ‘non‐cognitive processes’ contributing to social inequalities in education. It extends previous research highlighting discrepancies between student and teacher reported measures of such student traits (Desimone et al. 2010; Duckworth and Yeager 2015) by showing that the discrepancies are associated with student SES. This implies that research on social stratification in education needs to take particular care when including teacher or student reports of such student characteristics to mitigate over‐ or underestimation of SES influences. We also make a critical contribution to the scholarship that shows how congruence in teacher and student perceptions matters for effective teaching and learning (e.g., Könings et al. 2014) because our findings reveal how dissonance in perceptions is related to student SES. This may exacerbate social inequalities in teaching approaches and learning environments. Because teacher expectations shape students' feelings about school, academic self‐concept, confidence, aspirations and attainment (Papageorge et al. 2018; Wang et al. 2018), teacher perceptions that are positively ‘skewed’ towards social groups that are already experiencing advantages in school and negatively ‘skewed’ towards disadvantaged groups will strengthen social inequalities in education (see Francis et al. 2020). A socially stratified mismatch between teacher and student perceptions also contributes to inequalities in education because large perceptual discrepancies and discrepancies where teachers have more negative perceptions than the students likely represent teacher‐student relationships characterised by misunderstandings and conflict. Teacher‐student relationships in turn influence student outcomes and well‐being, especially for young and ‘at‐risk’ children (Hamre and Pianta 2001; McCormick et al. 2017). Another interesting finding of our study relates to student gender. Although not the focus of this paper, it is worth noting that for girls the perceptual discrepancies with teachers are significantly more advantageous than for boys.

The presented study has limitations which also highlight avenues for future research. What our findings cannot tell is who is ‘right’ and who is ‘wrong’. This is not a major drawback because, contrary to the typical teacher bias literature, we do not intend to compare a subjective teacher judgement to an objective measure, but rather to examine mismatches between two *subjective* perceptions—that of teachers and that of students. However, our perception measures may be affected by unconscious biases and motivational processes that are related to student SES and make teachers and students *report* perceptions that are ‘erroneous’. Teacher reports may be tainted by social class‐related stereotypes and unconscious biases, and low SES students' effort reports may be affected by social desirability or social enhancement desires. Through including prior ability into our analysis, we may be accounting for some of this skewedness but simultaneously making our student visibility measure, which is based on ability and SDQ, less robust because it may be affected by social desirability/self‐enhancement motivations. Another, related limitation of our study is that we cannot tell what other mechanisms drive the association between SES and teacher‐student perceptual discrepancies. Qualitative data on students' subjective framing and evaluation of effort and enjoyment in comparison to teachers' views and evaluation approaches as well as direct measures of students' social desirability/self‐enhancement desires and teachers' perception of student visibility could solve these ambiguities and fruitfully complement our findings.

Although our study cannot directly disentangle all the potential mechanisms leading to mismatches between teacher and student perceptions, they clearly show that children from socially disadvantaged backgrounds have perceptions of their enjoyment and effort that significantly deviate from their teachers' views, and that this deviation is to their detriment. Our study thus provides insights important for policy and practice. The findings suggest that creating conditions in which misunderstandings and conflict can be mitigated while close, positive teacher‐student communication and relationships are promoted could decrease SES‐influenced discrepancies between teacher and student perceptions. Student visibility may be an important key to reducing perceptual discrepancies. The day‐to‐day hustling and buzzing in classrooms, busyness of overstretched teachers, and myriad of needs and skills of students creates environments in which certain students become ‘invisible’ and, therefore, disadvantaged. These conditions could be altered through targeted contents in initial teacher education and teacher professional development aimed at promoting teachers' active formation of positive relationships with their students, professional self‐efficacy and skills in social perspective taking (Hajovsky et al. 2020; Robinson 2022). Scholars and policy makers concerned with understanding and tackling social inequalities in education should also take care to consider both teachers' *and* children's views and reports and pay particular attention to sociocultural and institutional circumstances shaping teacher stereotypes and unconscious biases, students' understanding of schools' expectations, and teacher‐student interactions.

## Ethics Statement

This research has received approval from the ethics committee of the authors' institution.

## Conflicts of Interest

The authors declare no conflicts of interest.

## Supporting information


Supporting Information S1

## Data Availability

The data that support the findings of this study are openly available in UKDS at https://ukdataservice.ac.uk/.

## Endnotes

^1^
Recent psychological research discussing the mismatch between working‐class and school cultures focuses for example on values around independence and cooperation (Goudeau et al. 2025; Yılmaz et al. 2024).

^2^
The only exception is the question ‘How much do you like school?’ asked to students in England. Here, answers are coded into a 3‐point scale: *A lot, A bit, Not at all*.

^3^
While the technique we use to calculate the teacher‐student perceptual discrepancies (see Section 2.2) redresses this imbalance to some extent, it may have implications because higher SES students and girls, for example, tend to be more interested in and have higher academic self‐concepts in language‐related domains than lower SES students and boys.

^4^
This last category includes children living in families with only unemployed and/or inactive parent(s).

^5^
To check whether a more recent ability measure would change results, we ran additional analyses using, for both England and Scotland, ability at age 11. The results are nearly identical to those using the age 5 measures.

^6^
The correlation test operationalises teacher perceptions in Scotland using the constructed index.

^7^
The correlation test operationalises teacher perceptions in Scotland and student perceptions in both England and Scotland using the constructed indices.

## Appendix

**TABLE A1:** OLS regression of teacher perception on student perception, school enjoyment.

	Teacher's perception	
	England	Scotland
Student's perception	0.275*** (0.016)	0.223*** (0.032)
Constant	0.000 (0.016)	0.000 (0.028)
*N*	5430	1682
R^2^	0.075	0.050
*F*‐test	F (1, 5428) = 298.15***	F (1, 1680) = 49.62***

+*p* < 0.10.

**p* < 0.05.

***p* < 0.01.

^***^ *p* < 0.001.

**TABLE A2:** OLS regression of teacher perception on student perception, school effort.

	Teacher's perception	
	England	Scotland
Student's perception	0.253*** (0.016)	0.221*** (0.035)
Constant	0.000 (0.015)	0.000 (0.029)
*N*	5477	1701
R^2^	0.064	0.049
*F*‐test	F (1, 5475) = 259.52 ***	F (1, 1699) = 41.14 ***

+*p* < 0.10.

**p* < 0.05.

***p* < 0.01.

^***^ *p* < 0.001.

**TABLE A3:** OLS regression of residuals on student characteristics, school enjoyment.

		England		Scotland	
		M1	M2	M1	M2
Social class	NS‐SEC 1, 2	0.000	0.000	0.000	0.000
		(.)	(.)	(.)	(.)
	NS‐SEC 3	−0.027	0.006	−0.179*	−0.080
		(0.043)	(0.041)	(0.079)	(0.076)
	NS‐SEC 4	−0.270***	−0.165**	−0.159	−0.134
		(0.059)	(0.056)	(0.100)	(0.094)
	NS‐SEC 5	−0.276***	−0.128+	−0.337**	−0.280*
		(0.077)	(0.075)	(0.106)	(0.112)
	NS‐SEC 6, 7	−0.318***	−0.169***	−0.358***	−0.259**
		(0.052)	(0.050)	(0.094)	(0.093)
	Not working	−0.431***	−0.215***	−0.665***	−0.421**
		(0.052)	(0.051)	(0.137)	(0.133)
GENDER	Boy		0.000		0.000
			(.)		(.)
	Girl		0.323***		0.329***
			(0.030)		(0.054)
SDQ			−0.242***		−0.185***
			(0.018)		(0.034)
BAS II naming vocabulary			0.053**		0.062*
			(0.018)		(0.029)
BAS II picture similarities			−0.004		0.066*
			(0.015)		(0.030)
CONSTANT		0.162***	−0.076**	0.181***	−0.038
		(0.020)	(0.026)	(0.032)	(0.043)
*N*		5430	5430	1682	1682
R^2^		0.032	0.127	0.045	0.129
*F*‐test: model		F (5, 5424) = 22.01***	F (9, 5420) = 51.14***	F (5, 1676) = 8.63***	F (9, 1672) = 16.85***
*F*‐test: main SES effect		F (5, 5424) = 22.01***	F (5, 5420) = 6.51***	F (5, 1676) = 8.63***	F (5, 1672) = 4.07**

+*p* < 0.10.

^*^ *p* < 0.05.

^**^ *p* < 0.01.

^***^ *p* < 0.001.

**TABLE A4:** OLS regression of residuals on student characteristics, school effort.

		England		Scotland	
		M1	M2	M1	M2
Social class	NS‐SEC 1, 2	0.000	0.000	0.000	0.000
		(.)	(.)	(.)	(.)
	NS‐SEC 3	−0.035	−0.004	−0.074	0.021
		(0.045)	(0.042)	(0.079)	(0.074)
	NS‐SEC 4	−0.267***	−0.155**	0.059	0.057
		(0.058)	(0.055)	(0.087)	(0.082)
	NS‐SEC 5	−0.332***	−0.156*	−0.047	−0.052
		(0.081)	(0.075)	(0.120)	(0.109)
	NS‐SEC 6, 7	−0.279***	−0.124**	−0.303**	−0.226*
		(0.049)	(0.046)	(0.094)	(0.092)
	Not working	−0.399***	−0.177***	−0.558***	−0.362**
		(0.050)	(0.049)	(0.142)	(0.135)
GENDER	Boy		0.000		0.000
			(.)		(.)
	Girl		0.447***		0.469***
			(0.029)		(0.055)
SDQ			−0.230***		−0.211***
			(0.019)		(0.033)
BAS II naming vocabulary			0.073***		0.006
			(0.017)		(0.030)
BAS II picture similarities			0.002		0.000
			(0.016)		(0.030)
CONSTANT		−0.154***	−0.148***	0.113***	−0.162***
		(0.020)	(0.025)	(0.032)	(0.044)
*N*		5477	5477	1701	1701
R^2^		0.028	0.148	0.034	0.144
*F*‐test: model		F (5, 5471) = 20.64***	F (9, 5467) = 70.12***	F (5, 1695) = 5.19***	F (9, 1691) = 16.79***
*F*‐test: main SES effect		F (5, 5471) = 20.64***	F (5, 5467) = 4.87***	F (5, 1695) = 5.19***	F (5, 1691) = 2.89**

+*p* < 0.10.

^*^ *p* < 0.05.

^**^ *p* < 0.01.

^***^ *p* < 0.001.
