# Supplementary material for: Student Socioeconomic Status and Teacher‐Student Perceptual Discrepancies of School Effort and Enjoyment
Source: Br J Sociol. 2025 Oct 1;77(1):52–73. doi: 10.1111/1468-4446.70035 (PMC12793720; doi:10.1111/1468-4446.70035)

**Supplementary materials for the paper ‘Student socioeconomic status and teacher-student perceptual discrepancies of school effort and enjoyment’**

This document presents results of three robustness analyses. In the first one we replace our main SES-measure (NS-SEC) with mother’s education. In the second analysis we apply a method of ‘simple differences’ to capture teacher-student perceptual discrepancies instead of the residual approach. The third analysis excludes the gender variable in order to check whether it affects our test of the student visibility-mechanism captured through prior ability and SDQ.

1. **Using mother’s education as SES measure**

For this analysis maternal education – as collected from the MCS and the GUS – is collapsed into the 7 categories presented in Table S1.

Table S1: maternal education categories (NVQ)

|  | **ACADEMIC QUALIFICATIONS** | **VOCATIONAL QUALIFICATIONS** |
| --- | --- | --- |
| **NVQ LEVEL 5** | Higher degree and postgraduate qualifications | Professional qualifications at degree level - e.g., graduate member of professional institute, chartered accountant or surveyor |
|  | Post-graduate Diplomas and Certificates |  |
| **NVQ LEVEL 4** | First degree (including B.Ed.) | Nursing or other medical qualifications (below degree level) |
|  | Diplomas in higher education and other higher education qualifications | NVQ or SVQ level 4 or 5 |
|  | Teaching qualifications for schools or further education (below degree level) | HND, HNC, Higher Level BTEC/RSA Higher Diploma |
| **NVQ LEVEL 3** | A/AS/S Levels/SCE Higher, Scottish Certificate Sixth Year Studies, Leaving Certificate or equivalent | NVQ or SVQ Level 3/GNVQ Advanced or GSVQ Level 3 |
|  |  | OND, ONCM BTEC National, SCOTVEC National Certificate |
|  |  | City & Guilds advanced craft, Part III/RSA Advanced Diploma |
| **NVQ LEVEL 2** | O Level or GCSE grade A-C, SCE Standard, Ordinary grades 1-3 or Junior Certificate grade A-C | NVQ or SVQ Level 2/GNVQ Intermediate or GSVQ Level 2 |
|  |  | BTEC, SCOTVEC first or general diploma |
|  |  | City & Guilds Craft or Part II/RSA Diploma |
|  |  | Other (older) trade apprenticeships |
| **NVQ LEVEL 1** | CSE below grade 1 / GCSE or O Level below grade C, SCE Standard, Ordinary grades below grade 3 or Junior Certificate below grade C | NVQ or SVQ Level 1/GNVQ Foundation Level or GSVQ Level 1 |
|  |  | BTEC, SCOTVEC first or general certificate/SCOTVEC modules |
|  |  | City & Guilds part 1/RSA Stage I, II, III/Junior certificate |
| **OVERSEAS QUALIFICATIONS** | Other academic qualifications (including overseas) | Other vocational qualifications (including overseas) |
| **NONE OF THESE** | None of these qualifications | None of these qualifications |

The variable maternal education is coded as presented in Table S2.

Table S2: coding of maternal education variable

| Maternal education 1 | NVQ-equivalent Level 5 |
| --- | --- |
| Maternal education 2 | NVQ-equivalent Level 4 |
| Maternal education 3 | NVQ-equivalent Level 3 |
| Maternal education 4 | NVQ-equivalent Level 2 |
| Maternal education 5 | NVQ-equivalent Level 1 |
| Maternal education 6 | Other / Overseas qualifications |
| Maternal education 7 | None of these |

Table S3: change in MATERNAL EDUCATION GAP across models, SCHOOL ENJOYMENT

|  | ENGLAND | | | SCOTLAND | | |
| --- | --- | --- | --- | --- | --- | --- |
|  | M1 | M2 | M3 | M1 | M2 | M3 |
| MATERNAL EDUCATION GAP (1-5) | .31 (.08) *** | .13 (.08) *+* | .13 (.09) | .42 (.14) ** | .26 (.13) + | .27 (.13) * |
| Δ from M1 |  | -.18 (.03) *** | -.18 (.05) *** |  | -.17 (.05) *** | -.15 (.07) * |
| *in percent* |  | 58.5 | 59.2 |  | 39.8 | 36.3 |
| MATERNAL EDUCATION GAP (1-7) | .44 (.09) *** | .20 (.08) * | .17 (.11) | .62 (.15) *** | .41 (.16) ** | .65 (.18) *** |
| Δ from M1 |  | -.24 (.04) *** | -.27 (.08) *** |  | -.21 (.06) *** | .03 (.09) |
| *in percent* |  | 53.8 | 62.3 |  | 33.3 | 5.1 |

*+p<0.10, * p<0.05, ** p<0.01, *** p<0.001*

*NOTE: the MATERNAL EDUCATION GAP is reported as b(SE)*

Table S4: Change in MATERNAL EDUCATION GAP across models, SCHOOL EFFORT

|  | ENGLAND | | | SCOTLAND | | |
| --- | --- | --- | --- | --- | --- | --- |
|  | M1 | M2 | M3 | M1 | M2 | M3 |
| MATERNAL EDUCATION GAP (1-5) | .43 (.08) *** | .24 (.08) ** | .28 (.09) ** | .36 (.15) * | .24 (.14) + | .28 (.14) * |
| Δ from M1 |  | -.19 (.03) *** | -.15 (.06) ** |  | -.11 (.05) * | -.08 (.08) |
| *in percent* |  | 44.2 | 34.6 |  | 31.5 | 22.2 |
| MATERNAL EDUCATION GAP (1-7) | .45 (.08) *** | .20 (.08) * | .20 (.11) + | .45 (.17) ** | .35 (.16) * | .53 (.18) ** |
| Δ from M1 |  | -.25 (.04) *** | -.25 (.07) ** |  | -.10 (.06) + | .09 (.10) |
| *in percent* |  | 54.8 | 54.7 |  | 22.3 | 19.7 |

*+p<0.10, * p<0.05, ** p<0.01, *** p<0.001*

*NOTE: the MATERNAL EDUCATION GAP is reported as b(SE)*

Figure S1: Average linear prediction of teacher bias by maternal education, SCHOOL ENJOYMENT


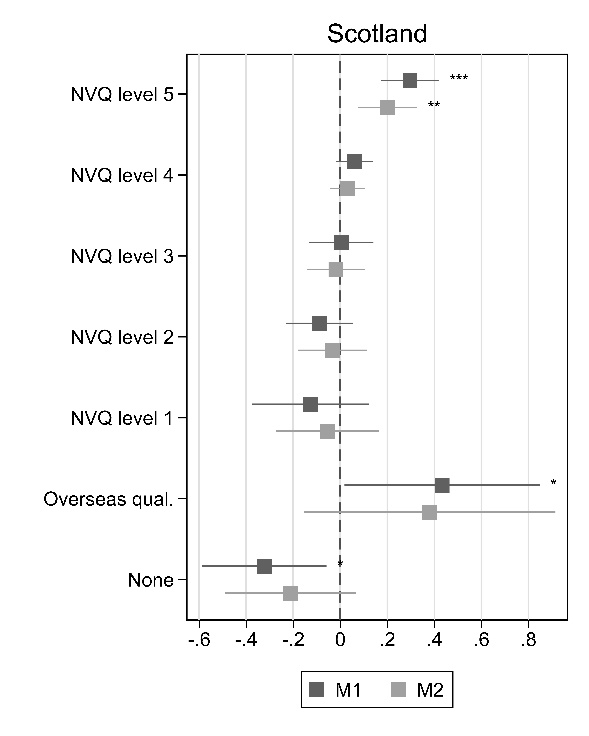

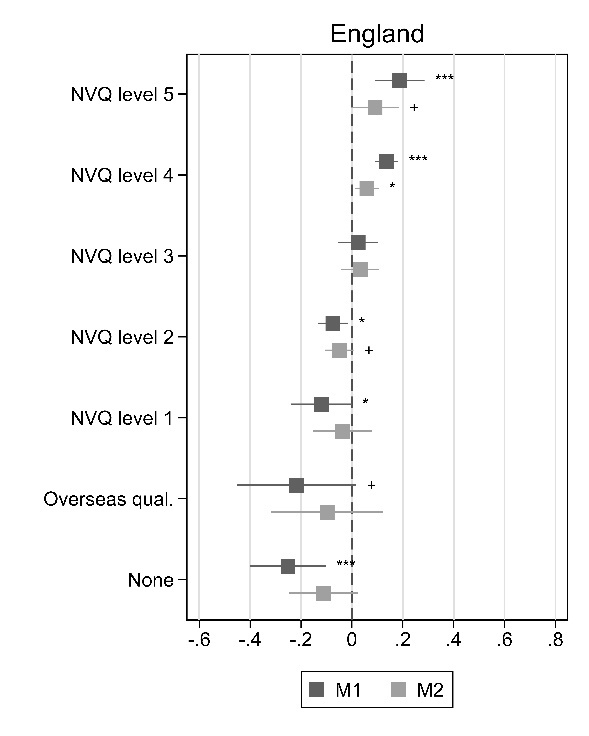


*+p<0.10, * p<0.05, ** p<0.01, *** p<0.001*


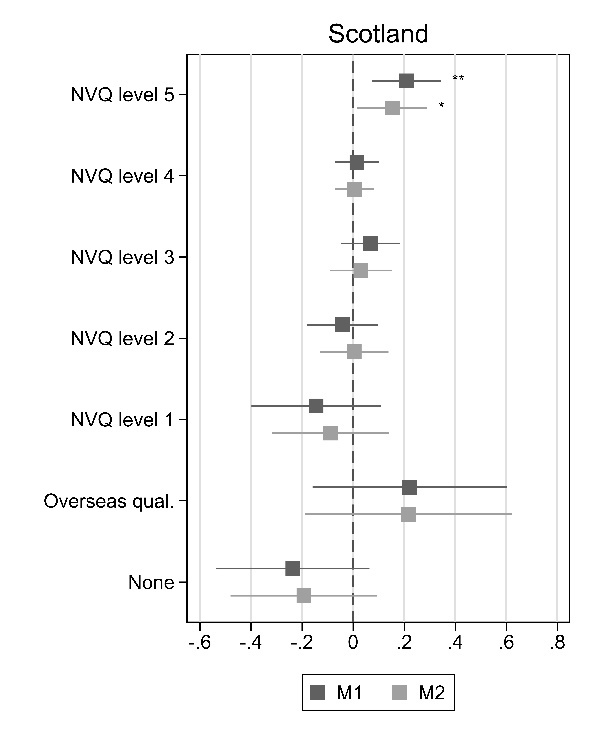
Figure S2: Average linear prediction of teacher bias by maternal education, SCHOOL EFFORT


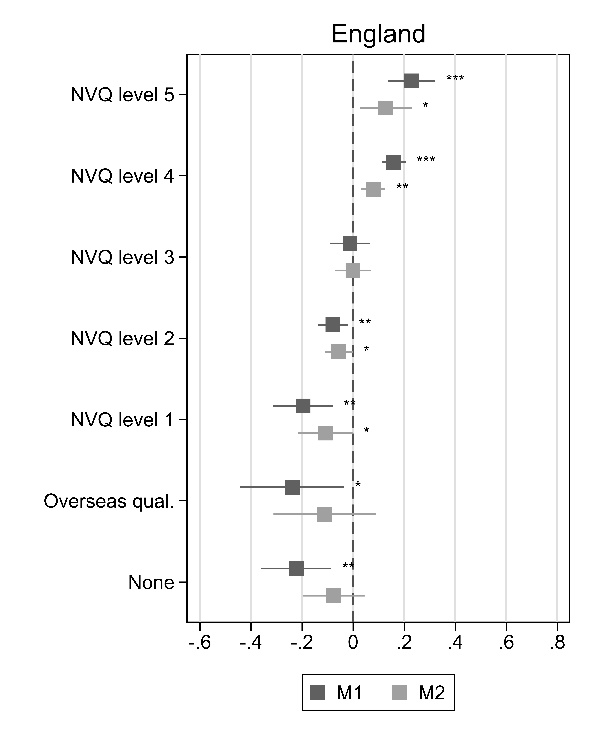


*+p<0.10, * p<0.05, ** p<0.01, *** p<0.001*

1. **Using ‘simple differences’ to capture teacher-student perceptual discrepancies**

This robustness check replaces residuals with a difference between the (std) teacher report and the (std) student report. The difference is then standardised again and treated as a continuous variable when predicting discrepancies in the final sets of models.

Table S5: Change in SOCIAL CLASS GAP across models, SCHOOL ENJOYMENT

|  | ENGLAND | | | SCOTLAND | | |
| --- | --- | --- | --- | --- | --- | --- |
|  | M1 | M2 | M3 | M1 | M2 | M3 |
| SOCIAL CLASS GAP (1-5) | .17 (.05) ** | .11 (.05) * | .12 (.06) * | .24 (.10) * | .23 (.10) * | .24 (.10) * |
| Δ from M1 |  | -.06 (.01) *** | -.05 (.02) ** |  | -.02 (.02) *** | -.01 (.03) ** |
| *in percent* |  | 34.3 | 30.8 |  | 7.6 | 3.5 |

*+p<0.10, * p<0.05, ** p<0.01, *** p<0.001*

*NOTE: the SOCIAL CLASS GAP is reported as b(SE)*

Table S6: Change in SOCIAL CLASS GAP across models, SCHOOL EFFORT

|  | ENGLAND | | | SCOTLAND | | |
| --- | --- | --- | --- | --- | --- | --- |
|  | M1 | M2 | M3 | M1 | M2 | M3 |
| SOCIAL CLASS GAP (1-5) | .20 (.05) *** | .12 (.05) ** | .12 (.05) ** | .18 (.09) + | .14 (.09) | .14 (.10) |
| Δ from M1 |  | -.08 (.01) *** | -.08 (.02) *** |  | -.04 (.02) + | -.04 (.03) |
| *in percent* |  | 41.4 | 39.7 |  | 19.9 | 23.2 |

*+p<0.10, * p<0.05, ** p<0.01, *** p<0.001*

*NOTE: the SOCIAL CLASS GAP is reported as b(SE)*

Figure S3: Average linear prediction of teacher bias by social class, SCHOOL ENJOYMENT


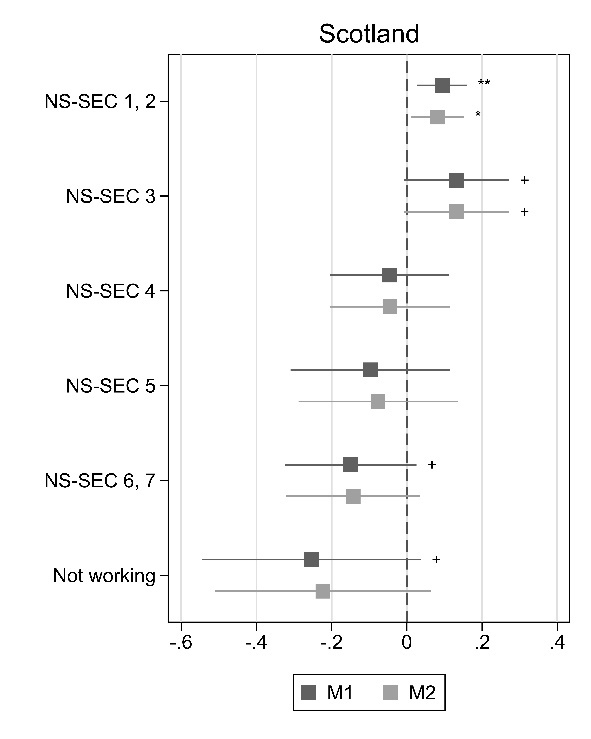

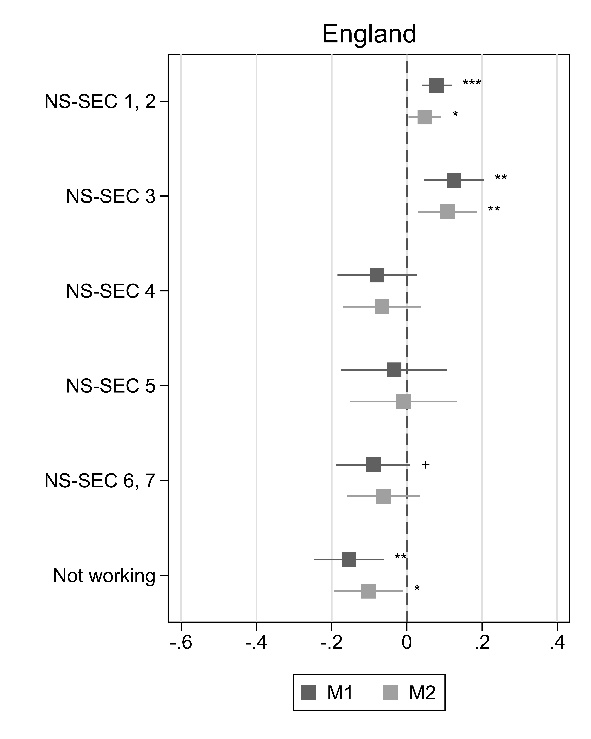


*+p<0.10, * p<0.05, ** p<0.01, *** p<0.001*


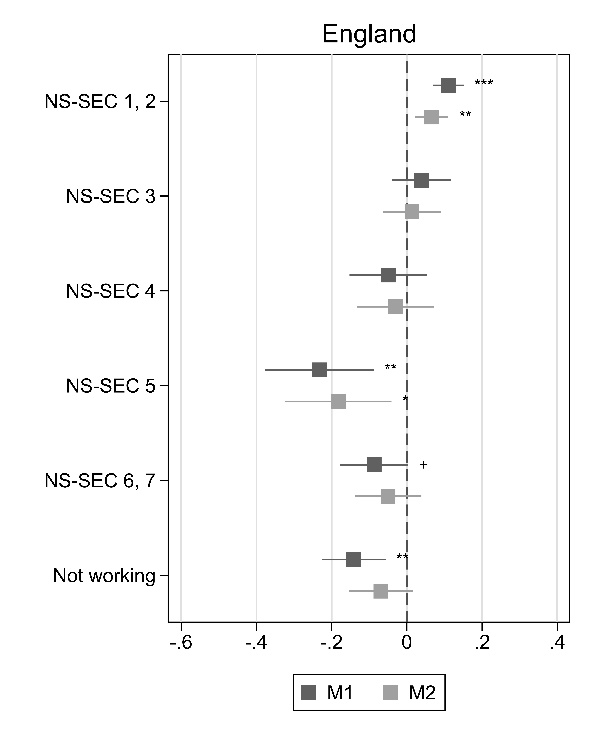
Figure S4: Average linear prediction of teacher bias by social class, SCHOOL EFFORT


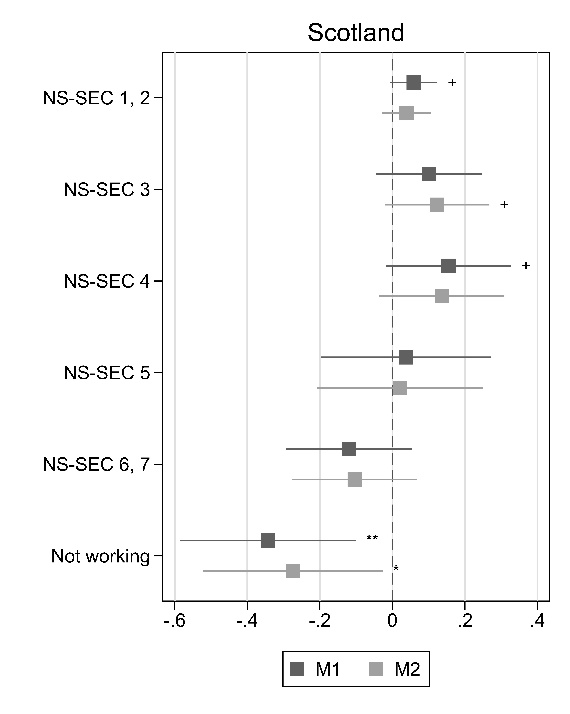


*+p<0.10, * p<0.05, ** p<0.01, *** p<0.001*

1. **Excluding gender**

This robustness analysis shows the regression models excluding gender in order to check whether *simultaneously* including gender, prior ability and SDQ affects the test of the student-visibility mechanism. The labelling in Figures S5 and S6 follows Table S5 above.

Table S7: Change in SOCIAL CLASS GAP across models, SCHOOL ENJOYMENT

|  | ENGLAND | | | SCOTLAND | | |
| --- | --- | --- | --- | --- | --- | --- |
|  | M1 | M2 |  | M1 | M2 |  |
| SOCIAL CLASS GAP (1-5) | .32 (.05) *** | .16 (.05) ** |  | .36 (.09) *** | .25 (.09) ** |  |
| Δ from M1 |  | -.15 (.02) *** |  |  | -.11 (.03) *** |  |
| *in percent* |  | 48.4 |  |  | 30.9 |  |

*+p<0.10, * p<0.05, ** p<0.01, *** p<0.001*

*NOTE: the SOCIAL CLASS GAP is reported as b(SE)*

Table S8: Change in SOCIAL CLASS GAP across models, SCHOOL EFFORT

|  | ENGLAND | | | SCOTLAND | | |
| --- | --- | --- | --- | --- | --- | --- |
|  | M1 | M2 |  | M1 | M2 |  |
| SOCIAL CLASS GAP (1-5) | .28 (.05) *** | .12 (.05) * |  | .30 (.09) ** | .21 (.09) * |  |
| Δ from M1 |  | -.16 (.02) *** |  |  | -.09 (.03) *** |  |
| *in percent* |  | 58.6 |  |  | 31.1 |  |

*+p<0.10, * p<0.05, ** p<0.01, *** p<0.001*

*NOTE: the SOCIAL CLASS GAP is reported as b(SE)*

Figure S5: Average linear prediction of teacher-student perceptual discrepancies by student SES, school enjoyment


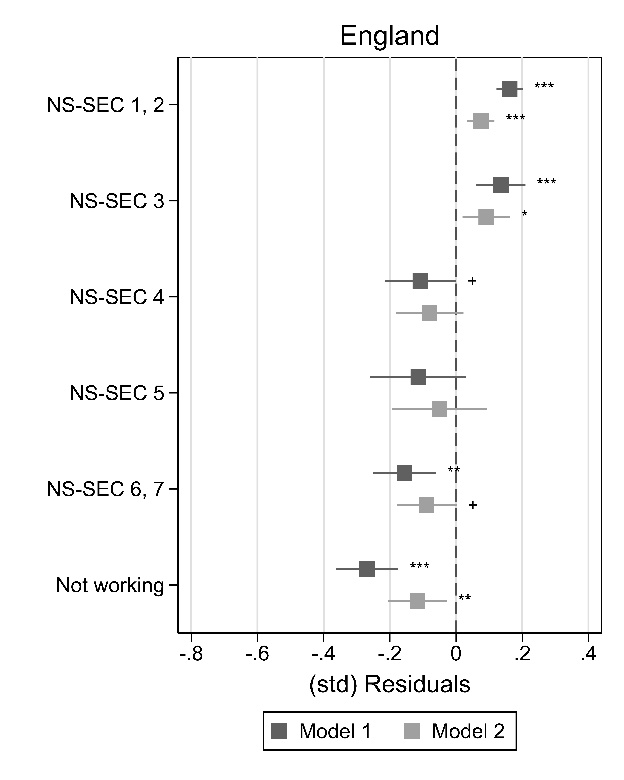

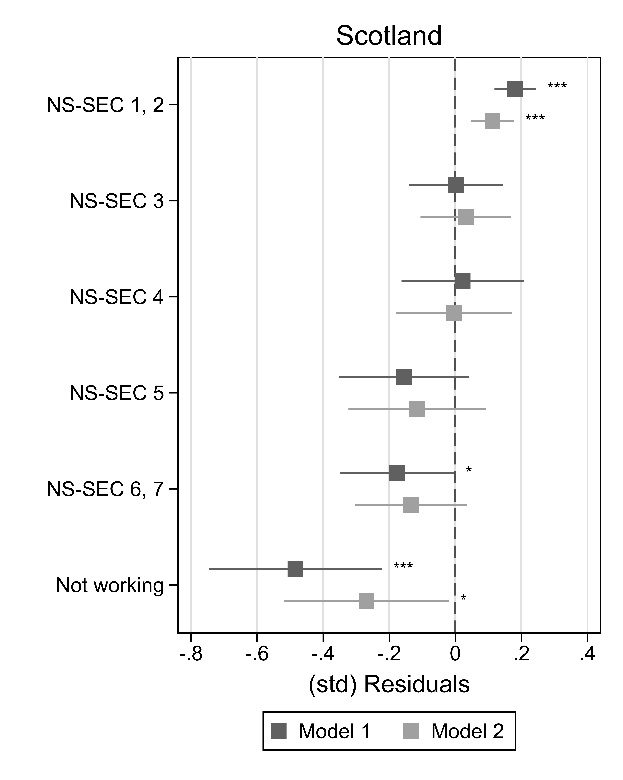


Figure S6: Average linear prediction of teacher-student perceptual discrepancies by student SES, school effort


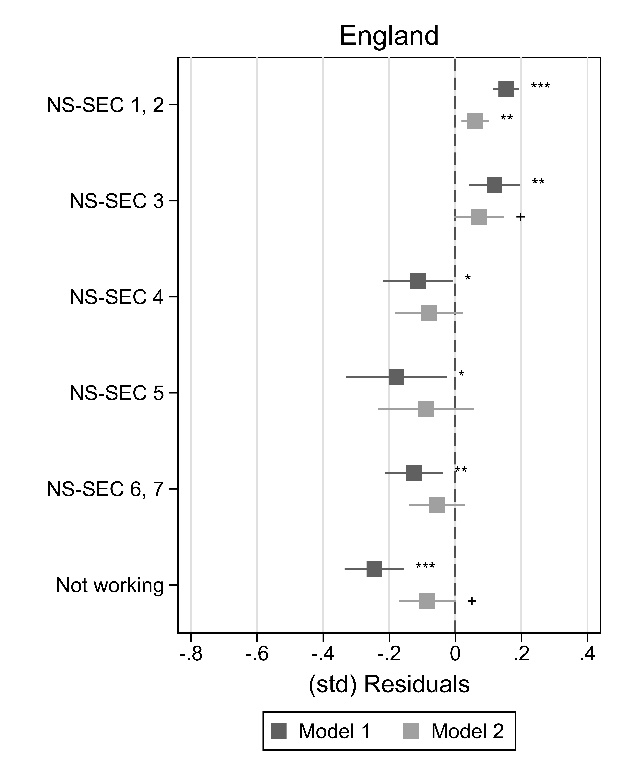

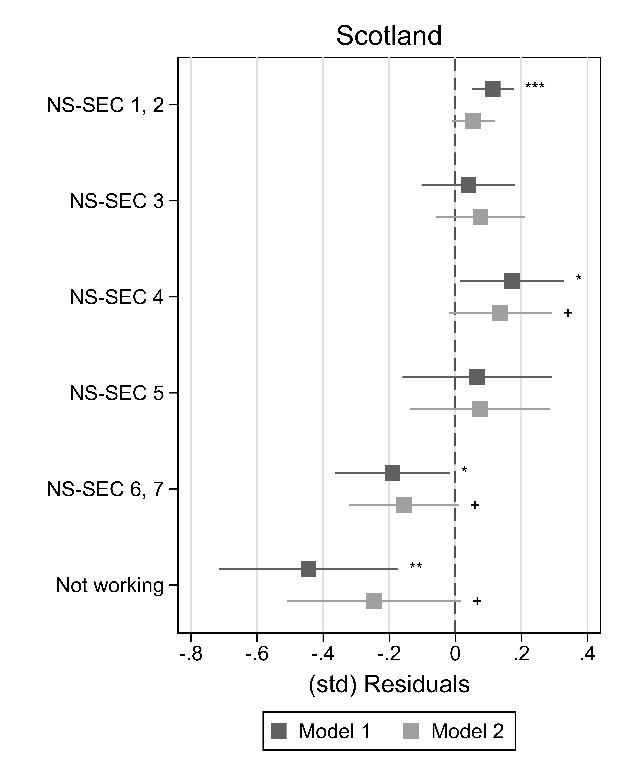

Supplement: Supplementary file 1 — Supporting Information S1 [file BJOS-77-52-s001.docx]
